# Evaluation of Convolutional Neural Networks (CNNs) in Identifying Retinal Conditions Through Classification of Optical Coherence Tomography (OCT) Images

**DOI:** 10.7759/cureus.77109

**Published:** 2025-01-07

**Authors:** Rohin R Teegavarapu, Harshal A Sanghvi, Triya Belani, Gurnoor S Gill, K.V. Chalam, Shailesh Gupta

**Affiliations:** 1 Department of Science, American Heritage High School, Delray Beach, USA; 2 Department of Technology and Clinical Trials, Advanced Research, Deerfield Beach, USA; 3 Chester F. Carlson Center for Imaging Science, Rochester Institute of Technology, Rochester, USA; 4 Department of Medicine, Florida Atlantic University Charles E. Schmidt College of Medicine, Boca Raton, USA; 5 Department of Ophthalmology, Loma Linda University, Loma Linda, USA; 6 Department of Ophthalmology, Broward Health North, Deerfield Beach, USA

**Keywords:** artificial intelligence, cnn, diabetic retinopathy, machine learning, ophthalmology

## Abstract

Introduction

Diabetic retinopathy (DR) is a leading cause of blindness globally, emphasizing the urgent need for efficient diagnostic tools. Machine learning, particularly convolutional neural networks (CNNs), has shown promise in automating the diagnosis of retinal conditions with high accuracy. This study evaluates two CNN models, VGG16 and InceptionV3, for classifying retinal optical coherence tomography (OCT) images into four categories: normal, choroidal neovascularization, diabetic macular edema (DME), and drusen.

Methods

Using 83,000 OCT images across four categories, the CNNs were trained and tested via Python-based libraries, including TensorFlow and Keras. Metrics such as accuracy, sensitivity, and specificity were analyzed with confusion matrices and performance graphs. Comparisons of dataset sizes evaluated the impact on model accuracy with tools deployed on JupyterLab.

Results

VGG16 and InceptionV3 achieved accuracy between 85% and 95%, with VGG16 peaking at 94% and outperforming InceptionV3 (92%). Larger datasets improved sensitivity by 7% and accuracy across all categories, with the highest performance for normal and drusen classifications. Metrics like sensitivity and specificity positively correlated with dataset size.

Conclusions

The study confirms CNNs’ potential in retinal diagnostics, achieving high classification accuracy. Limitations included reliance on grayscale images and computational intensity, which hindered finer distinctions. Future work should integrate data augmentation, patient-specific variables, and lightweight architectures to optimize performance for clinical use, reducing costs and improving outcomes.

## Introduction

Many studies have been conducted on machine learning (ML) algorithms specifically trained to diagnose retinal conditions [1,2]. As advancements were made in this field of ML research specifically, researchers have explored the utility of efficiently and accurately automating the process of diagnosing retinal conditions [3]. Past studies have shown that ML algorithms are mostly accurate when providing adequate information, almost matching diagnoses by medical professionals specializing in ophthalmology [4]. However, these studies show that these models are best suited for a secondary role, working with medical professionals’ diagnoses [5]. A review of past studies indicates that different ML-based architectures, when augmented with additional patient health data, still need to be evaluated to improve the accuracy of the correct diagnosis of retinal conditions [6].

Diabetic retinopathy (DR): diagnosis and concerns

DR is a non-curable complication of diabetes in which blood vessels in the retina are damaged [7]. High blood sugar in the body can damage the retina and cause this condition, hence the name “diabetic retinopathy.” Early signs of DR, found through eye scans, include hemorrhages and microaneurysms in the retinal blood vessels and solidified exudates, masses of cells, and fluid being released [8]. Early on, DR patients are asymptomatic [8]. Later symptoms include blurred vision, dark areas in vision, impaired color vision, and vision loss in more extreme cases [9]. Over seven million people in the United States have been diagnosed with DR, and it is the leading cause of blindness on a global scale [9].

DR can lead to a variety of other severe eye conditions, the first of which is diabetic macular edema (DME). DME is caused by the leaking of exudates, a symptom of DR [9]. This is the cause of unclear or “blurry vision.” Some fluids must leave the eye, and DR can cause blood vessels to grow out of the retina and block these fluids, leading to neovascular glaucoma, which can cause blindness. Other side conditions include cataracts and retinal detachment [9].

As DR is a widespread retinal condition, especially in the United States, tens of millions of dollars have been spent on advanced optometry equipment. The average day-one expenditure total for the required technology is over $200,000, according to Miki Lyn Zilnicki, OD (Doctor of Optometry) [10].

DR is diagnosed by ophthalmologists using a variety of techniques. These include visual acuity tests, tonometry, and pupil dilation eye exams [11]. However, there is a level of subjectivity in diagnosing someone with any condition/condition related to DR. This can lead to misdiagnosis in the form of false positives (FPs) or false negatives (FNs) [11]. ML algorithms can counteract this subjectivity by training these algorithms with eye scans/images and reinforcing the accuracy of DR diagnoses [12]. A portion of said expenditures are passed to consumers as fees and other required payments, increasing due to economic and technological factors. Eye exam fees have increased by over 190% from 1985 to 2015, from $50 to $145 [13].

ML applications for DR diagnosis

DR is diagnosed by ophthalmologists using a variety of techniques. These include visual acuity tests, tonometry, and pupil dilation eye exams. However, there is a level of subjectivity in diagnosing someone with any condition or condition related to DR [11]. This can lead to misdiagnosis in the form of FPs or FNs. ML algorithms can counteract this subjectivity by training these algorithms with eye scans/images and reinforcing the accuracy of DR diagnoses [11].

The backbones of medical research are practicality and efficiency. In ophthalmology, ML algorithms are practical and efficient, with the additional trait of cost-effectiveness [14]. Lim et al. conducted a study that utilized the EyeArt Artificial Intelligence System to detect cases of DR from participants using fundus photography imaging. The participants underwent two-field fundus photography, dilated ophthalmoscopy, and four-widefield stereoscopic dilated fundus photography. A total of 521 participants (999 eye images) successfully met the criteria and had their retinal images used in the study, out of 893 that chose to participate [15]. The study concluded that the EyeArt system, in some aspects, more accurately came to the correct conclusion and diagnosis compared to general ophthalmologists or retina specialists [15]. The study also concluded that artificial intelligence systems could be a cheaper alternative to expensive eye scans and eye scanning technology [15].

Padhy et al. reviewed the current state of AI in diagnosing DR and other retinal conditions and disorders utilizing fundus photographs of retinal scanning. The review concluded that using AI was a “boon” for ophthalmologists and patients and that integrating artificial intelligence in ophthalmology would curtail costs and optimize visits per doctor [16]. However, the study warned that AI should not be used as a substitute for medical professionals in the field of ophthalmology; instead, it should be used in conjunction with ophthalmologists [16]. There is no shortage of research involving the application of machine-learning algorithms in medical research, but no such techniques have been introduced or explored at a large scale. As such, there is a heightened uncertainty surrounding ML and its capabilities, especially for its recency relative to existing ophthalmologic technology [16].

This idea of uncertainty was explored by Rajesh et al. in their analysis of the abilities of artificial intelligence. They referenced previous studies, and in their conclusions, they addressed the need for more knowledge to utilize artificial intelligence properly [17]. They also state the importance of creating and identifying superior algorithms to utilize as time passes and state that the variability of costs in the different regions of the world and specific healthcare systems dramatically affects our ability to quantify the cost-effectiveness of ML algorithms definitively [17].

However, this does not mean estimates have not been made. Even leaders in modern technology, such as Google, have had teams of experts discovering solutions to diagnose DR early [18]. Their mission was to develop an algorithm that could identify abnormalities in retinal scans from a hospital in India, a nation with over 18 million already suffering from DR [18]. It has taken its toll, especially on those under the poverty line, not able to take care of themselves or seek medical attention before it is far too late for anything of significant value to happen. Identifying the condition early would save the vision of millions to come, and Varun Gulshan, a research scientist from Google, was appointed for the project [19]. They developed an application that utilized the ARDA algorithm or Automated Retinal Disease Assessment. This program assisted in speeding up the rate of ophthalmologists treating DR by filtering out those who do not have DR currently and leaving only those with diagnoses to visit an ophthalmologist and receive treatment. The project was a resounding success for Google and the team appointed to it [19].

Convolutional neural networks (CNNs) are a class of neural networks in ML used for solving computer vision-related issues [20]. These algorithms can classify visual data (i.e., digital images) by identifying patterns and features [20]. Advancements in convolution-based ML have occurred for over 42 years since its inception. Considering their ability to recognize visual patterns across all types of visual data, CNNs have seen widespread use in the medical field, specifically to identify anomalies and predict onsetting conditions [21]. Computer vision has incorporated itself into many aspects of everyday life and the economy through machine-learning-powered solutions in various fields [22].

This study uses two architectures, VGG16 and InceptionV3, to develop and evaluate an ML-based model, specifically a CNN. These models will be trained to identify features in retinal image data to facilitate efficient and accurate diagnosis of various eye conditions. The study will leverage ML algorithms, images obtained from portable retinal imaging devices, and open-source software to create adaptive models. This approach reduces the time and expenses of diagnosing benign and malignant retinal conditions during comprehensive eye examinations.

In addition, the tool developed in this research will assist medical professionals in making informed decisions based on visual information and patient-related data. The project involves training and testing an algorithm to interpret and classify retinal images by identifying distinct features and categorizing the data accordingly. At peak performance, the ML-based algorithm is expected to nearly rival the diagnostic abilities of medical professionals specializing in retinal conditions. The project’s results will likely lead to more studies exploring ML applications within the field of ophthalmology.

## Materials and methods

This study uses four classes depicting different retinal conditions using images to train CNNs, as depicted in Table 1.

**Table 1:** Description of different retinal conditions CNV, choroidal neovascularization; DME, diabetic macular edema

Classes	Description
Normal retina	No conditions or abnormalities
CNV	Condition related to new blood vessels causing damage to the eye
DME	Condition related to eye linked to complications of diabetes
Drusen	Condition based on spots in the eye representing age-related macular degeneration

**Figure 1:**
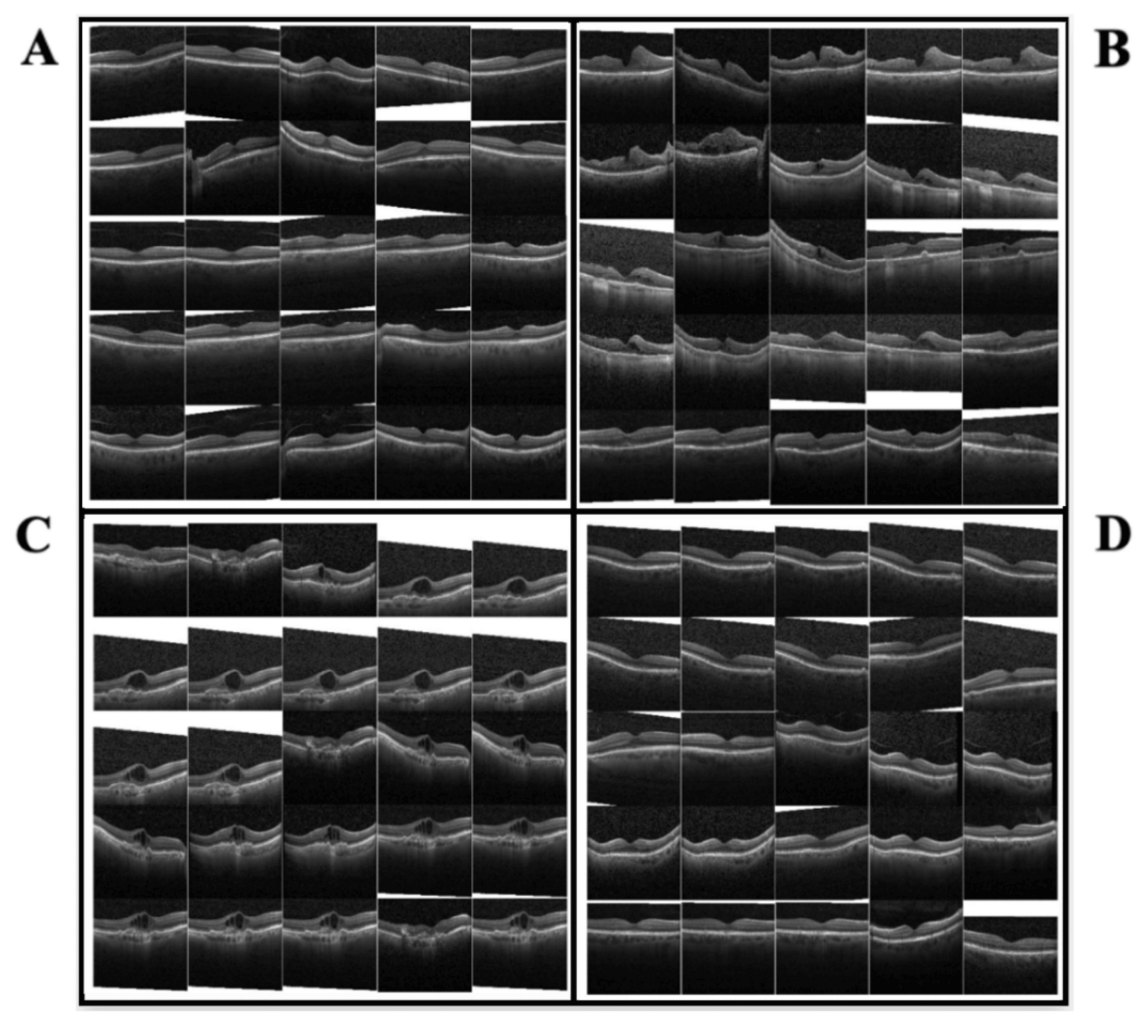
Classification of retinal conditions: examples of OCT images used to train the dataset OCT, optical coherence tomography

Additionally, there should be a positive correlation between the number of training images in a dataset and the accuracy of the training and testing on the said dataset. Additional information in the dataset (patient data and vitals) should improve the ML algorithm’s classification abilities of the four conditions, as shown in Figure 1.

Figure 1A represents standard retinal images with no abnormalities. Figure 1B shows images of DME, characterized by fluid accumulation and swelling in the macula, often due to complications of diabetes. Figure 1C illustrates choroidal neovascularization (CNV), marked by the growth of abnormal blood vessels beneath the retina, which can lead to significant vision loss. Figure 1D depicts drusen, yellow deposits beneath the retina commonly associated with age-related macular degeneration (AMD).

CNN architecture

**Figure 2:**
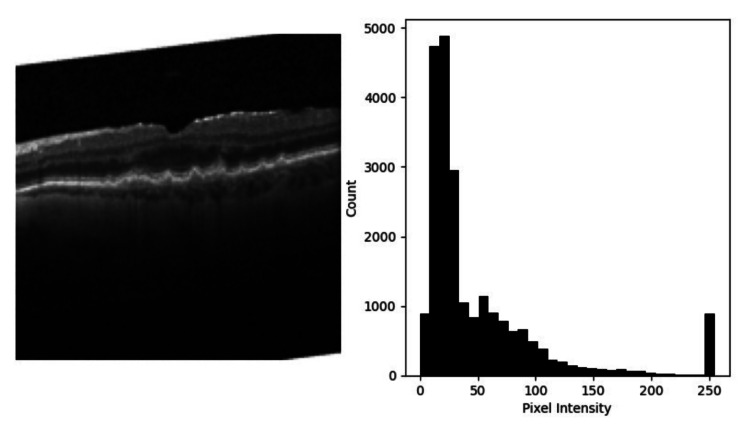
Pixel intensity distribution and structural analysis of retinal OCT image example OCT, optical coherence tomography

As shown in Figure 2, the CNN architecture comprises multiple neuron layers, beginning with the input neuron layer, each representing a particular pixel in any image. Their activation depends entirely on pixel intensity and directly influences the following layers through connected weights and biases. Hidden convolution layers are tasked with finding specific patterns. These patterns may or may not be readable by humans. Still, weights and biases connected to previous layers can change the patterns each neuron is associated with, as each neuron is associated with multiple neurons in previous layers by weights. Each neuron is tasked to find these patterns, along with more weights and biases. These convolutional layers are split by max-pooling layers, which aim to reduce the number of neurons by extracting only the most essential features [22]. Once the activation of the output layer is determined, each value is put through a nonlinearity function, such as a ReLU or SoftMax function, that condenses the activation of a said neuron to a value between 0 and 1. This is essential for calculating confidence values and, by extension, accuracy and cost metrics. Cost metrics have a significant role, as minimizing this metric is the heart of ML [23-25]. No additional data augmentation techniques, such as image rotation, flipping, cropping, or color adjustments, were applied to the dataset in this study, as the focus was solely on evaluating the performance of predefined CNN architectures (VGG16 and InceptionV3) on the original optical coherence tomography (OCT) image dataset.

Application to case study

This study requires a device compatible with a terminal, generally a computer or laptop. A terminal will be required to install the required Python packages locally and launch the needed software. This device should have the following minimum specifications 12 GB RAM for running the program using the entire dataset, 12 GB of available storage for the dataset, Python and other package installations, and other miscellaneous files, and Windows 10/MacOS Catalina 10.15.7/Linux (Ubuntu 16.04) to use JupyterLab. For example, cost-effective choices include the Dell OptiPlex 7040 and Lenovo ThinkPad E1 ($350-$500). Memory requirements can be surpassed by utilizing virtual memory, but this requires additional available storage space.

Dataset

The images are derived from publicly available datasets online, which is why an IRB was not used for this study [26,27]. Furthermore, according to the information provided by these sources, the images were de-identified and consent was obtained. Additionally, in order to utilize these images, Python 3.12.4 must be installed from python.org with administrator permission to PATH (both checkboxes are in the bottom left corner in the first section of the setup file). Next, use the command prompt to install the following libraries: NumPY, MatPlotLib, Glob, SciKit-Image, SciKit-Learn, OpenCV, Pandas, Seaborn, ZLib, IterTools, SciPY, CSV, TQDM, Keras, TensorFlow, JupyterLab, PIL, and ImbalancedLearn. These can all be installed as specific commands utilizing pip, Python’s dedicated package installation software. The installation commands can be found online at each package’s respective website.

Once Python and all relevant packages are installed, launch JupyterLab through the terminal using the command “jupyter lab”. Do not close the terminal, as it hosts your kernel, which is required to run programs in JupyterLab. Then, upload the newest version of the program to JupyterLab, after which the server will request a kernel to be used, in which case Python 3 (ipykernel) should be used. After this, the program can be executed using the double arrow icon on the top when the kernel is idle. The status of the kernel is identified on the top right side of the program ribbon. The program will require up to two hours to execute fully. Should there be no error at any point during the program, the last cell will produce as its output the results of said program, which includes a table of calibration and validation accuracies by epoch, three different graphical representations of said table, and a confusion matrix that details specific accuracies by category across all epochs.

## Results

The following sections present results and observations from multiple experiments using CNNs with different architectures from the Python-based ML API Keras (i.e., VGG16 and InceptionV3) to classify OCT images. The architectures of VGG16 and InceptionV3 differ in the number of hidden layers in CNN. After the ML-based models using two architectures are executed, graphs and tables are obtained, each with a series of metrics. Inferences drawn from the results related to classification accuracies and observations are provided. The charts and tables provided details on the performance of two different model architectures across multiple training cycles with varying training datasets of different sizes.

The values in Figure 1 show the CNN model’s ability to learn and improve from previous epochs; however, this is only true for training data. The model can learn to make proper predictions to a certain extent. Still, additional learning begins to stagnate around epochs 4-6, shown in Figure 1A and 1B, graphical forms of the table in Figure. 1. An epoch refers to a training cycle when all the images are presented to the CNN once. Also, loss metrics seem almost inversely proportional to accuracy. Still, the values that define these two metrics are not the same, so this relationship is circumstantial.

**Figure 3:**
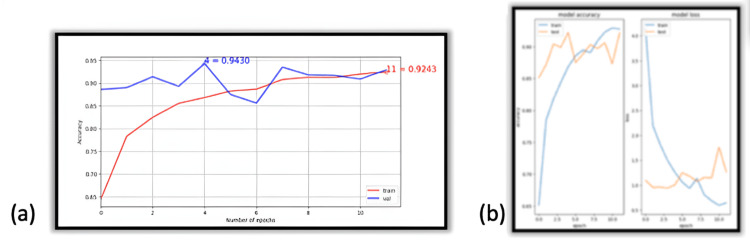
VGG16 performance metrics: accuracy and loss trends across training epochs (a) Accuracy by epoch and (b) accuracy and loss metrics for the VGG16 architecture using the entire dataset.

The graphs in Figure 3A and 3B are similar to those in Figure 1 but convey different conclusions. The training dataset sizes can affect the model’s performance on the testing data, the size of which stays the same throughout. The performance between dataset sizes differs by at most 8% based on the dataset size. VGG16 also improved similarly when the dataset size was expanded.

**Figure 4:**
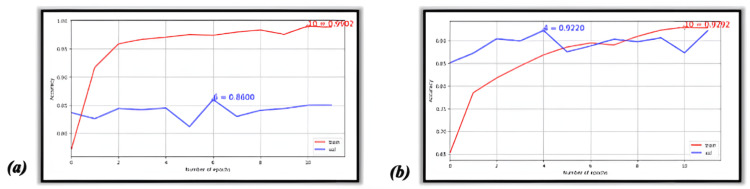
Peak accuracy by epoch for InceptionV3 architecture (a) 4,000 images and (b) full dataset

Figure 4 provides a detailed spread of correct and incorrect classifications, commonly called the multi-class confusion matrix, which details the occurrence of events based on the predictions the model makes compared to the actual class the images belong to. For n classes, n2 values exist on the matrix, with each row representing the model’s predictions and each column representing the actual class for each image. For example, the cell in row 1 and column 1 represents the number of occurrences where the model correctly classified an image belonging to the normal class as part of the normal class..

**Figure 5:**
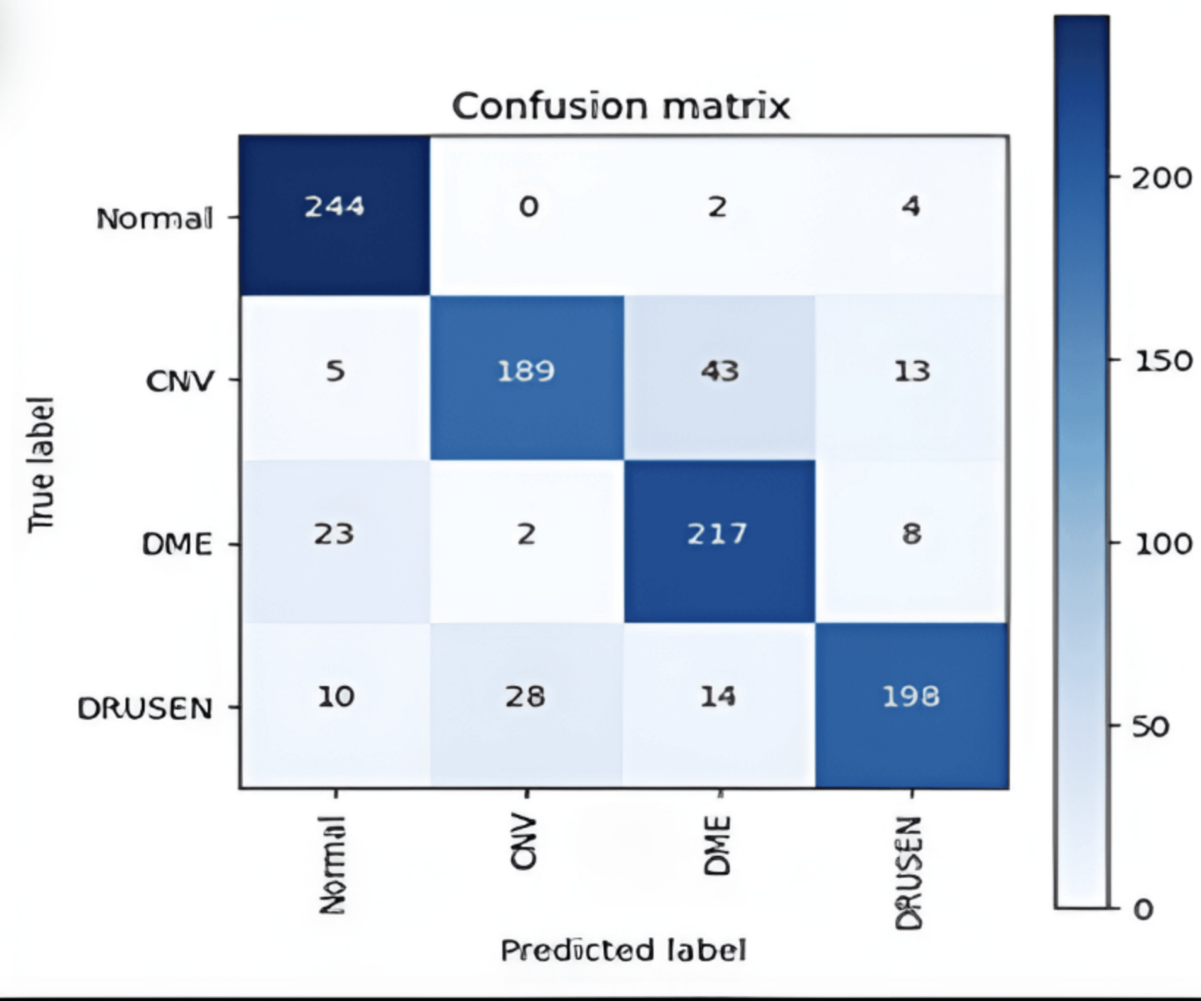
Confusion matrix of results

With the confusion matrix in Figure 5, we can find the following metrics: true positive (TP), true negative (TN), FP, and FN. However, these metrics are obtained through a similar two-confusion matrix with positive and negative rows and columns. As such, when finding these metrics with multi-class confusion matrices, each class in said confusion matrix has specificity and sensitivity metrics. For example, the TP is the number of occurrences of an image of the normal class correctly predicted as such, but TNs are the number of occurrences of an image not of the normal predicted to not be of the normal class (not necessarily a correct prediction). Details of the specificity and sensitivity metrics for the full and small datasets (i.e., 1,000 images) are provided in Table 2 and Table 3.

Additional metrics

**Table 2:** Performance metrics for VGG16 architecture CNV, choroidal neovascularization; DME, diabetic macular edema

Image class	Performance metric					
	Accuracy	Specificity	Sensitivity	Precision	Recall	F1 score
Normal	0.9627	0.9703	0.9387	0.92	0.94	0.9267
CNV	0.9437	0.9767	0.8453	0.92	0.8467	0.8967
DME	0.9317	0.9503	0.875	0.8933	0.8733	0.88
Drusen	0.9367	0.9523	0.8907	0.8633	0.89	0.8767

**Table 3:** Performance metrics for InceptionV3 architecture CNV, choroidal neovascularization; DME, diabetic macular edema

Image class	Performance metric					
	Accuracy	Specificity	Sensitivity	Precision	Recall	F1 score
Normal	0.9687	0.987	0.9133	0.96	0.91	0.9367
CNV	0.9407	0.964	0.8707	0.8933	0.8733	0.88
DME	0.954	0.9683	0.9093	0.9067	0.9067	0.9067
Drusen	0.925	0.9393	0.8827	0.83	0.8833	0.8533

With these metrics, specificity (TN rate) and sensitivity (TP rate) metrics can be obtained for each class. The specificity formula is TP/(TP + FN), and the sensitivity formula is TN/(TN + FP). As such, these metrics are related to accuracy such that an increase in accuracy is likely to correlate to an overall rise in specificity and sensitivity, as only some niche scenarios that are almost impossible to exist under normal conditions can break this correlation. Dataset size, which has a similar correlation to accuracy, is another that can improve these metrics. For VGG16, the smallest data set size used was around 4,000 images. For comparison, the whole dataset contains 83,000 images. This difference in dataset size led to a 2% average increase in specificity and a 5% average increase in sensitivity.

With this information, three other relevant metrics can be found using the confusion matrix. Each class will also have its own four metrics. The first metric is the OR (TP + TN/FP + FN), which measures the chance of any image classified into some class N belonging to class N. The second metric is the bias score (TP + FP)/(TP + FN), a ratio of how many images were classified into some class N compared to how many are a part of class N. The third and final metric is the probability of false detection (FP/TN + FP), a measure of the chance that an image not in class N is incorrectly classified into class N.

## Discussion

Comparison to other studies

Bhandari et al. developed a more efficient, lightweight CNN model containing 983,716 parameters, incorporating explainable AI techniques like LIME and SHAP for interpretation. They also tested their model’s generalizability on other medical imaging datasets, including COVID-19 and kidney stones, demonstrating its adaptability beyond retinal conditions, achieving an average accuracy of 94.29% [28]. Their study analyzed additional metrics such as precision, recall, F1-score, and ROC curve, with an AUC of 0.99. Their 10-fold cross-validation demonstrated consistent accuracy, with 96.33% training accuracy and 94.12% validation accuracy, highlighting the model's balanced performance across different classes, including drusen and CNV. The results of Bhandari et al. align with our study in demonstrating that CNNs can effectively classify retinal conditions [28]. However, their higher average accuracy and use of a more detailed evaluation, including precision (0.94-0.95) and recall (0.90-0.96), suggest that their lightweight CNN model may be more efficient and robust [28].

Regarding model architecture, our study used complex, parameter-heavy models (VGG16 and InceptionV3), which were computationally intensive while achieving high accuracy (up to 94%). This complexity could limit real-time clinical applicability and make the models susceptible to overfitting. In contrast, Hassan et al. used a modified ResNet-50 architecture, known for its deep residual layers and ability to reduce training time while maintaining accuracy [29]. By combining ResNet-50 with a random forest algorithm and utilizing dual optimizers, they achieved an impressive accuracy of 97.47%. This performance outperformed other models, including VGG16 and InceptionV3, indicating a more robust approach to classification [29].

Arefin et al. also focused on retinal disease classification but developed a non-transfer deep CNN model tailored for OCT images, achieving a higher accuracy of 97.9%. Their results supported using CNNs for retinal classification, similar to our study. Still, they highlighted that a non-transfer learning approach could outperform traditional transfer learning models like VGG-19 (93.3%) and InceptionV3 (95.8%), the latter being one of the architectures we used [30]. Arefin et al. conducted a detailed post hoc analysis, revealing that only eight out of 256 filter kernels in their model's final convolutional layer contributed to classification [30]. This suggests simpler models might be as effective as more complex ones. This finding does not directly contradict our study but extends it by indicating that models designed explicitly for retinal OCT images can achieve higher accuracy and efficiency [30].

Puneet et al. employed a transfer learning approach similar to ours, using VGG16 for retinal disease classification, but with critical enhancements that contributed to their success [31]. They introduced an attention mechanism within the model to focus on the most relevant features in the OCT images, improving its ability to differentiate complex retinal abnormalities [31]. Additionally, they used data augmentation techniques, such as rotation, flipping, and cropping, to expand the training dataset, which likely prevented overfitting and enhanced the model's generalizability [31].

Their study achieved a testing accuracy of 95.6%, slightly higher than the 94% peak accuracy we obtained using VGG16 [31]. Puneet et al. also conducted a more thorough evaluation by reporting metrics like precision, recall, F1-score, and ROC curves, providing a comprehensive performance assessment that our study needed to include [31]. Integrating an attention module improved feature extraction, helping the model concentrate on critical areas within the retinal images for better diagnostic accuracy. Puneet et al.’s findings indicate that adding attention mechanisms and data augmentation could enhance our model’s performance. Furthermore, their detailed evaluation approach suggests incorporating additional metrics into our study would give a clearer picture of the model’s diagnostic capabilities [31].

Another study by Singh et al. proposes a custom CNN model based on VGG16 with additional convolutional layers tailored specifically for retinal diseases. The model reaches an impressive 98% accuracy [32]. The added layers enhance performance in classifying cataracts, glaucoma, and DR, proving highly effective in distinguishing these diseases [32].

Singh et al. and our study leverage large datasets of retinal images, yet they differ in their approach to image preprocessing. Our study uses grayscale images, while Singh et al. retain color, likely aiding disease differentiation as color provides richer detail for pattern recognition [32]. This difference in preprocessing might account for the higher accuracy in Singh et al., as color variations can highlight unique features associated with each retinal condition [32]. Additionally, Singh et al. employ data augmentation techniques, such as rotation and flipping, contributing to the model’s robustness by exposing it to diverse image transformations. These techniques likely improve the model’s generalization abilities and guard against overfitting, an issue we encounter in our study with slight oscillations in validation loss across epochs due to architectural complexity. Performance metrics further distinguish the two studies [32].

Our study focuses on classification accuracy and sensitivity across four categories, noting that dataset size has a positive effect, with larger datasets slightly improving accuracy and sensitivity metrics [32]. In contrast, Singh et al. evaluated precision, recall, and F1 scores for each disease, achieving high sensitivity and specificity [32]. By tailoring layers to particular diseases, this study quantifies model performance in greater detail, reinforcing its higher accuracy and resilience against challenges such as class imbalance [32]. These performance metrics emphasize the value of disease-specific layer customization in enhancing diagnostic precision.

Limitations and error analysis

One of the biggest challenges of the study was developing additional metrics for a multi-class confusion matrix. Metrics like specificity and sensitivity needed to be calculated for each of the four classes, and technicalities for metrics like TNs caused issues regarding defining these metrics. The TN value for some class N in a multi-class confusion matrix is the instance wherein images not of class N are not predicted to be part of class N. This immediately produces a contradiction. For example, if these metrics are for the normal class, an image of class CNV predicted to be a part of class DME represents an incorrect classification. However, it would still be considered a TN, a correct classification, thus producing a contradiction. To resolve this, TNs still need to satisfy the previous condition for this study, but they also need to be a correct classification on our confusion matrix.

One limitation of the study lies in its reliance on grayscale OCT images, which may not fully capture the variability present in real-world datasets, potentially impacting the generalizability of the findings. Additionally, while class imbalances in the dataset were mitigated using weighted loss functions, this inherent characteristic of the data may still influence the performance metrics and their applicability to more balanced datasets. These same images in color could have allowed for a more prominent distinction between classes and could potentially increase accuracy, but this is hypothetical. Additionally, not all data available to a medical professional were provided to the models. Knowledge of vitals, preexisting conditions, and other statistics could have impacted accuracy. This could not be done correctly; however, because the models tested were CNNs, models pre-trained specifically on visual data, and as such, they are models that are primarily meant to analyze visual data. CNNs may not properly analyze textual data. While it is possible that these models could use said data meaningfully to influence their classifications, it is unknown.

Clinical impact and digital twin (DT)

The clinical implications of the study on CNNs in identifying retinal conditions align extensively with the potential of DT technology, particularly in predictive modeling, personalized disease management, surgical planning, real-time monitoring, resource optimization, and long-term disease management [33]. The CNN models demonstrated high accuracy in classifying retinal conditions like DR, CNV, and macular edema through OCT image analysis, and this diagnostic precision can be expanded within a DT framework by integrating a range of patient-specific variables such as genetic predispositions, vitals, and lifestyle factors [34]. In practice, DT models could utilize this comprehensive data to simulate the disease progression dynamically for individual patients, offering a real-time reflection of a patient's health status that facilitates continuous monitoring and the early identification of disease markers [35]. For instance, combining CNN’s disease classification capabilities with DT’s simulation of progression based on updated patient data makes implementing targeted interventions far earlier in the disease course possible [35].

DT technology further enhances personalization in managing diseases by enabling real-time simulation of how patients respond to various treatments, which could complement CNN’s diagnostic function [36-38]. With DT, clinicians can test and refine potential treatment pathways, such as anti-VEGF injections for macular edema, based on the patient-specific predictions generated by the CNN diagnostics. In surgical contexts, DT technology supports preoperative planning by providing a virtual “twin” of the patient, which allows surgeons to simulate and plan surgeries with a model customized to the patient’s anatomy and specific condition [39]. The ability of DT models to provide real-time updates and predictive analytics has substantial implications for monitoring and decision support in clinical practice. This dynamic, ongoing feedback loop ensures that healthcare providers are equipped with actionable insights and can adjust treatment protocols proactively to serve the patient’s evolving condition best [39].

Regarding cost and resource optimization within ophthalmology, the CNN study highlights substantial potential savings in diagnostic time and costs [40]. The integration of CNNs and DTs allows these efficiencies to be taken further by reducing the need for repeated, often costly, diagnostic tests. For example, instead of scheduling frequent OCT scans, a DT system could simulate disease progression by extrapolating data from existing scans and combining it with the patient’s additional health information, thereby maintaining precise monitoring without continuous testing [40]. This approach cuts costs for patients and healthcare facilities and improves the allocation of clinical resources by streamlining the care process.

Lastly, DT technology offers significant improvements for long-term disease management and outcome prediction, which is especially valuable in chronic conditions like AMD [41]. By integrating the diagnostic data from CNNs, clinicians can use DTs to simulate potential disease trajectories, accounting for lifestyle factors, genetic predispositions, and real-time updates [42-43]. These insights allow healthcare providers to anticipate a patient’s health outcomes over time, enabling proactive adjustments to treatment plans based on the evolving risk profile of the patient [43]. Through this integration, healthcare providers can establish a robust system that covers all stages of care, from the initial diagnosis to long-term management, ultimately supporting improved patient outcomes in a more efficient and personalized way.

## Conclusions

The CNN models adequately recognized patterns that clearly distinguish the other classes. Trained on the full dataset of images, the Keras model VGG16 obtained a peak accuracy of 94%, whereas the Keras model InceptionV3 obtained a similar peak accuracy of 92%. The accuracy rates of both models show that they can recognize common distinctions between classes, but finer details need to be accounted for. On a large scale, CNNs like the one tested in this study would play an important secondary role in ophthalmologists’ ability to validate diagnoses with a simple retinal image. For next to no cost, we could have a solution for funneling misdiagnosed patients away from expensive treatments and medical professional visits, cutting expenses, and saving time for patients and medical facilities. The medical field runs on efficiency, and ML’s applications in this field embody that very purpose. These models have yet to show their potential. Many factors critical to the models’ high accuracy were not expanded upon completely, leaving even more room for improvement despite their already exemplary performance. For example, this dataset is only a fraction of the size needed for future endeavors and the OCT images.
